# Cucurbitacin E Induces G_**2**_/M Phase Arrest through STAT3/p53/p21 Signaling and Provokes Apoptosis *via* Fas/CD95 and Mitochondria-Dependent Pathways in Human Bladder Cancer T24 Cells

**DOI:** 10.1155/2012/952762

**Published:** 2012-01-09

**Authors:** Wen-Wen Huang, Jai-Sing Yang, Meng-Wei Lin, Po-Yuan Chen, Shang-Ming Chiou, Fu-Shin Chueh, Yu-Hsuan Lan, Shu-Jen Pai, Minoru Tsuzuki, Wai-Jane Ho, Jing-Gung Chung

**Affiliations:** ^1^Department of Biological Science and Technology, China Medical University, Taichung 404, Taiwan; ^2^Department of Pharmacology, China Medical University, Taichung 404, Taiwan; ^3^Department of Functional Neurosurgery and Gamma Knife Center, China Medical University Hospital, Taichung 404, Taiwan; ^4^Department of Health and Nutrition Biotechnology, Asia University, Taichung 413, Taiwan; ^5^School of Pharmacy, China Medical University, Taichung 404, Taiwan; ^6^Department of Biochemistry, Nihon Pharmaceutical University, Saitama 362-0806, Japan; ^7^Tsuzuki Institute for Traditional Medicine, China Medical University, Taichung 404, Taiwan; ^8^Department of Medicinal Botanicals and Health Care, Da-Yeh University, Changhua 515, Taiwan; ^9^Department of Biotechnology, Asia University, Taichung 413, Taiwan

## Abstract

Cucurbitacin E, a tetracyclic triterpenes compound extracted from cucurbitaceous plants, has been shown to exhibit anticancer and anti-inflammatory activities. The purpose of this study was to elucidate whether cucurbitacin E promotes cell cycle arrest and induces apoptosis in T24 cells and further to explore the underlying molecular mechanisms. The effects of cucurbitacin E on T24 cell's growth and accompanied morphological changes were examined by MTT assay and a phase-contrast microscope. DNA content, mitochondrial membrane potential (ΔΨ_*m*_) and annexin V/PI staining were determined by flow cytometry. The protein levels were measured by Western blotting. Our results demonstrated that cucurbitacin E-induced G_2_/M arrest was associated with a marked increase in the levels of p53, p21 and a decrease in phospho-signal transducer and activator of transcription 3 (STAT3), cyclin-dependent kinase 1 (CDK1) and cyclin B. Cucurbitacin E-triggered apoptosis was accompanied with up-regulation of Fas/CD95, truncated BID (t-BID) and a loss of ΔΨ_*m*_, resulting in the releases of cytochrome *c*, apoptotic protease activating factor 1 (Apaf-1) and apoptosis-inducing factor (AIF), and sequential activation of caspase-8, caspase-9, and caspase-3. Our findings provided the first evidence that STAT3/p53/p21 signaling, Fas/CD95 and mitochondria-dependent pathways play critical roles in cucurbitacin E-induced G_2_/M phase arrest and apoptosis of T24 cells.

## 1. Introduction

In 2008, approximately 37500 new bladder cancer cases were diagnosed worldwide [1]. In the United States, approximately 68,810 people and 14,100 deaths from bladder cancer were reported in 2008 alone [1]. According to the Taiwan Department of Health annual reports 2009, bladder cancer is 14th leading cause of cancer death in Taiwanese men and about three persons per one hundred thousand die annually from that. Today, bladder cancer is usually treated with surgery, chemotherapy, and combination of chemotherapy and radiotherapy, but they are often intolerable because of the strong systemic toxicity and local irritation [2]. Therefore, the finding for a novel adjuvant agent to treat bladder cancer that can reduce the recurrence rate, decrease side effects, and increase overall survival is urgent. In the past ten years, investigators have focused on plant food-derived phytochemicals to find potential anticancer drugs [3, 4]. Approximately 70% of the currently approved anticancer drugs are derived from or based on natural products [5]. Cucurbitacin E (molecular formula: C_32_H_44_O_8_) is a natural flavonoid found in *Cucurbitaceae *but also presents in other plants [6–8] and traditional Chinese herbal medicine such as *Cucurbita pepo cv dayangua* [9]. Cucurbitacin E has been reported to exhibit biological activities, including antioxidant, anti-inflammatory, and anticancer effects [6, 9, 10]. Cucurbitacin E induced cytotoxic effects, including suppression of the proliferation and induction of apoptosis in ovarian [11, 12], leukemia [13, 14], and pancreatic [15] cancer cell lines. Cucurbitacin E also suppressed tumor angiogenesis through interacting vascular-endothelial-growth-factor-receptor-2-(VEGFR2-) mediated Janus kinase 2 (Jak2)-signal transducer and activator of transcription 3 (STAT3) signals [9].

It is well documented that apoptosis plays an important role in the maintenance of tissue homeostasis for the elimination of excessive cells [16]. However, it is also well known that the induction of apoptosis of cancer cells by anticancer drugs such as etoposide, cisplatin, and paclitaxel has been used for treatment of cancer in target cells [17–20]. Many reports have shown that numerous cytotoxic and DNA damaging agents could arrest the cell cycle at the G1, S, or G_2_/M phase and induce apoptotic cell death [21–23] which is involved in downregulation of phosphorylated STAT3, an oncogene that has a vital role at all stages of tumorigenesis [15]. Therefore, the present study investigated the induction of apoptosis by cucurbitacin E in human bladder cancer cells, and we also attempted to clarify the possible signaling pathways involved in cucurbitacin E-induced apoptosis. Our results indicated cucurbitacin E induced G_2_/M phase arrest and apoptosis in human bladder cancer T24 cells through STAT3/p53/p21, Fas/CD95, and mitochondria-dependent pathways.

## 2. Material and Methods

### 2.1. Chemicals and Reagents

Cucurbitacin E, dimethyl sulfoxide (DMSO), propidium iodide (PI), 3-(4,5-Dimethylthiazol-2-yl)-2,5-diphenyltetrazolium bromide (MTT), RNase A, and Triton X-100 were purchased from Sigma-Aldrich Corp. (St. Louis, MO, USA). All primary and secondary antibodies were obtained from Santa Cruz Biotechnology Inc. (Santa Cruz, CA, USA). The fluorescent probe DiOC_6_ and all culture media and reagents were purchased from Invitrogen Life Technologies (Carlsbad, CA, USA).

### 2.2. Cell Culture

The human bladder cancer cell line (T24) (transitional cell carcinoma) was purchased from the Food Industry Research and Development Institute (Hsinchu, Taiwan) and was cultured with McCoy's 5a medium supplemented with 10% FBS, 100 Units/mL penicillin, 100 *μ*g/mL streptomycin, and 2 mM L-glutamine at 37°C under a humidified 5% CO_2_ and 95% air at one atmosphere. The medium was changed every 2 days [24].

### 2.3. Determinations of Cell Morphology and Viability

About 2 × 10^5^ T24 cells/well were seeded into 12-well plates for cell adherence with three wells for each concentration level. Cucurbitacin E was individually added to the final concentrations of 0, 250, 500, 1000, and 2000 nM, respectively. In control group, equal amount of 1% DMSO was added, while in blank control group, only culture medium was added to each well. In the end of incubation for 24 or 48 h, cells from each well were examined and photographed under a phase-contrast microscope at 200× magnification and then cell viability was determined using the MTT method, as described previously [25, 26].

### 2.4. Cell Cycle Distribution and Phase Determination

The cell cycle distribution and sub-G1 phase were analyzed by flow cytometry, as described previously [27, 28]. A total of 2 × 10^5^ T24 cells/well on 12-well plates were incubated with 1000 nM cucurbitacin E or only with vehicle (DMSO, 1% in culture media) for 0, 12, 24, and 48 h. At the end of incubation, the cells on each well were trypsinized, washed with PBS, and fixed with 70% ethanol overnight at −20°C. At the next day, all fixed cells were from experimental and control groups washed twice with PBS and then were stained with PI-working solution (40 *μ*g/mL PI and 100 *μ*g/mL RNase A and 0.1% Triton X-100) for cellular staining at room temperature for 1 h in the dark. Then all cells were analyzed for cell cycle distribution and sub-G1 phase (apoptosis) by flow cytometry, as described previously [27, 28]. The fractions of the cells in G0/G1, S, and G_2_/M phase were analyzed using a FACSCalibur flow cytometer (Becton-Dickinson, Franklin Lakes, NJ, USA), and data were analyzed with a ModFit LT program (Verity Software House Inc., Topsham, ME, USA). 

### 2.5. Assessment of Apoptosis

Apoptotic cells were measured with an Annexin V-FITC/PI detection kit (Invitrogen Life Technologies). Approximately 2 × 10^5^ cells in 12-well plates per treatment or control samples (1000 nM cucurbitacin E for 12 and 24 h) were harvested and analyzed by flow cytometry, as described previously [26, 29]. Annexin V-FITC is a sensitive probe for identifying cells undergoing apoptosis as phosphatidylserine (PS) exposure occurs early in the apoptotic process. PI is excluded from live cells with intact plasma membranes, but it is incorporated into nonviable cells.

### 2.6. CDK1 Kinase Activity

T24 cells were seeded onto 75 cm^2^ tissue culture flask and then treated with 0, 250, 500, and 1000 nM of cucurbitacin E for 24 h. Cells were suspended in a final volume of 0.2 mL buffer containing 50 mM Tris-HC1 (pH 7.5), 1 mM phenylmethylsulfonyl fluoride, 50 pg/mL leupeptin, 10 mM 2-mercaptoethanol, 1 mM MgCl_2_, 2 mM EGTA, 0.5 mM dithiothreitol, 0.01% Brij35, 25 mM *β*-glycerophosphate, and 0.5 M NaCl. Cell suspensions were sonicated and centrifuged at 10,000 ×g for 30 min. CDK1 kinase activity condition was determined by using MV Peptide (CDK1 kinase assay kit, Medical & Biological Laboratories Co., Ltd., Nagoya, Japan) and measuring OD_492_, as described previously [26, 30].

### 2.7. Assay of Mitochondrial Membrane Potential (ΔΨ_*m*_)

A density of 2 × 10^5^ T24 cells/well on 12-well plates was maintained with 1000 nM cucurbitacin E for 0, 4, 8, 12, 24, and 48 h, to determine the level of ΔΨ_*m*_ by flow cytometry. At the end of incubation, cells were harvested, washed twice by PBS, and then resuspended in 500 *μ*L of DiOC_6 _(1 *μ*mol/l) for the level of ΔΨ_*m*_. Subsequently, cells were maintained at dark room for 30 min at 37°C and then all samples were analyzed immediately by flow cytometry, as described previously [31, 32].

### 2.8. Detection of Cell Viability after Pretreatment with a General Caspase Inhibitor

T24 cells at a density of 1 × 10^4^ cells/100 *μ*L seeded in 96-well plates were preincubated with or without 10 *μ*M of the general caspase inhibitor (Z-VAD-FMK, R&D Systems, Minneapolis, MN, USA) for 2 h before cells were exposed to 1000 nM cucurbitacin E for a 48-h exposure. Thereafter, cells were determined for the viability by using a MTT assay, as previously described [25, 26].

### 2.9. Western Blot Analysis for Protein Levels

T24 cells (1 × 10^6^/well) seeded into 6-well plates were treated with 1000 nM cucurbitacin E or solvent control. Cells were harvested from each treatment then were washed with cold PBS and then scraped and washed twice by centrifugation at 1,000xg for 5 min at 4°C. All pellets were individually resuspended in the PRO-PREP protein extraction solution (iNtRON Biotechnology, Seongnam, Gyeonggi-Do, Korea) for 3 h at −20°C, as described previously [33, 34]. The lysate from each sample was collected by centrifugation at 12,000 ×g for 30 min at 4°C, and the supernatant was stored at −20°C. Sodium dodecyl sulfate-polyacrylamide gel electrophoresis (SDS-PAGE) gels were used to separate the protein before each sample was incubated with the primary antibodies (Santa Cruz Biotechnology Inc.) followed by secondary antibodies. These blots were then detected by ECL kit (Millipore, Bedford, MA, USA) and autoradiography using X-ray film [27, 35]. Each PVDF membrane (Immobilon-P; Millipore) was stripped and reported with anti-*β*-actin antibody as the loading control for ensuring that equal proteins were loaded [32, 36].

### 2.10. Statistical Analysis

All values are expressed as mean ± standard deviation (S.D.) and performed in triplicate. One-way ANOVA was used to compare the difference between control and concentration treatments in each group followed by Bonferroni's multiple comparison tests. A *P* value of less than 0.05 was considered to be statistically significant.

## 3. Results

### 3.1. The Effects of Cucurbitacin E on Cell Growth and Viability of T24 Cells

**Figure 1:**
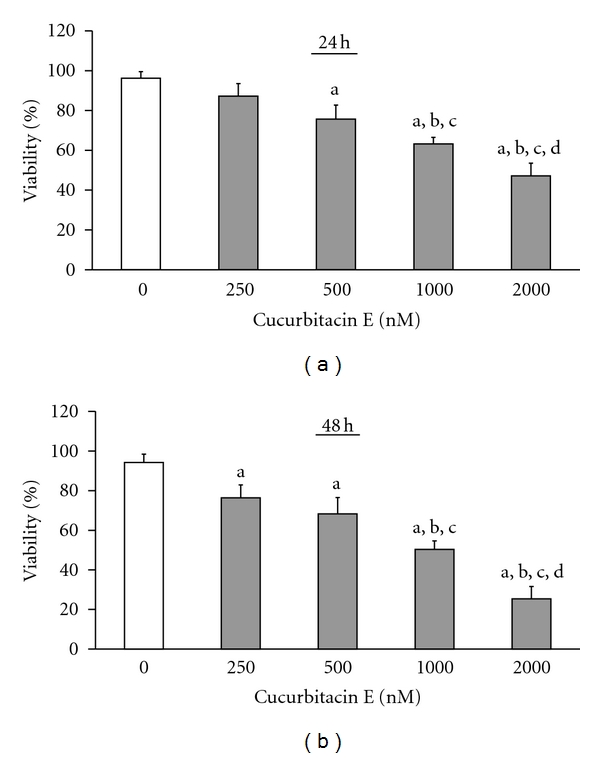
The chemical structure of cucurbitacin E and its effects of cell viability on human bladder cancer T24 cells. Cells were treated with different concentrations of cucurbitacin E for 24 h (a) and 48 h (b) and then cells were harvested for calculated the percentage of viable cells as described in the Materials and Methods. Data are presented as mean ± S.D. in triplicate. a, *P* ≤ 0.05, is significant different compared with the DMSO-treated control; b, c and d, *P* ≤ 0.05, are significant different with 250, 500 and 1000 nM of cucurbitacin E treatment, respectively, by one-way ANOVA followed by Bonferroni's test for multiple comparisons.

The examined concentrations of cucurbitacin E inhibited cell growth and induced cytotoxicity of T24 cells. Cells were treated with various concentrations (0, 250, 500, 1000, and 2000 nM) of cucurbitacin E or 1%, DMSO as a control for 24 and 48 h. Percentage of the live cells in each group was determined by MTT method. Our data indicated that cucurbitacin E at the concentrations of 250–2000 nM inhibited cell growth in a concentration- and time-dependent manner (Figures 1(a) and 1(b)) with the half maximal inhibitory concentration (IC_50_) of 1012.32 ± 10.6 nM after a 48 h treatment.

### 3.2. The Effects of Cucurbitacin E on DNA Content of T24 Cells

**Figure 2:**
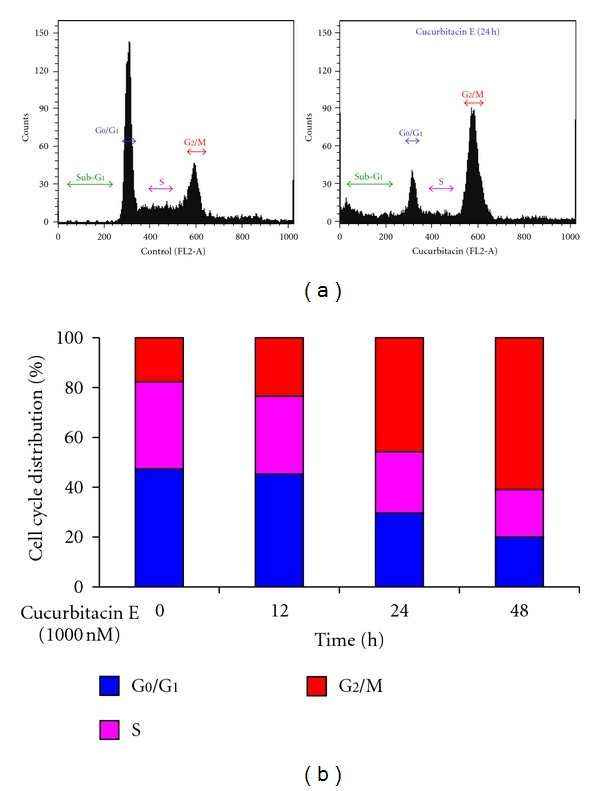
The effects of cucurbitacin E on the G_2_/M phase arrest in T24 cells. Cells at a density of 2 × 10^5^ cells per well were placed in 12-well plates and then were treated with 1000 nM cucurbitacin E for different time periods (12, 24, or 48 h). Cells were harvested for evaluating the cell cycle distribution as described in the Materials and Methods. (a) The representative of profiles of DNA content; (b) the percentage of cells in G0/G1, S, and G_2_/M phase in T24 cells. Data revealed a representative experiment in triplicate with similar results.

Cell-cycle distribution of T24 cells after treatment with 1000 nM cucurbitacin E for 12, 24, and 48 h was measured by flow cytometry. Results were shown that the profile from flow cytometry and BD CellQuest Pro software in the number of cells in G_2_/M phase was increased after treatment with 1000 nM of cucurbitacin E for 24 h (Figure 2(a)). The number of cells in G_2_/M phase was increased from 19.4% to 24.6%, 48.66% and 65.89% after 12, 24 and 48 h treatments, respectively and these effects are time-dependent responses (Figure 2(b)). These data suggest that the induction of G_2_/M phase arrest accounts for the growth inhibitory effects of cucurbitacin E-treated T24 cells.

### 3.3. The Effects of Cucurbitacin E on the Levels of STAT3, p53, and p21 as well as G_2_/M Phase-Associated Protein Levels in T24 Cells

**Figure 3:**
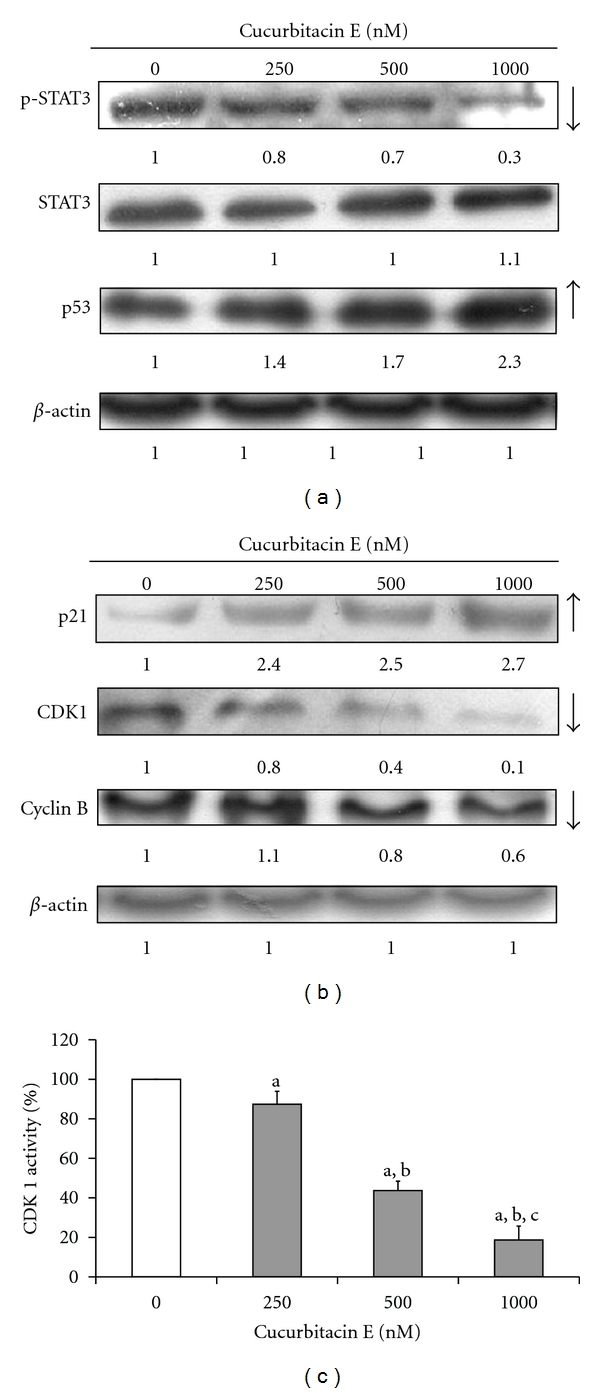
The effects of cucurbitacin E on the STAT3 phosphorylation, cell cycle-regulated associated proteins, and CDK1 activity in T24 cells. Cells at 1 × 10^6^ (cells/well) were placed in 6-well plates and then treated without and with 250, 500, and 1000 nM of cucurbitacin E for 24 h. Thereafter, cells were harvested and total proteins were collected for western blot analysis to examine the changes of protein levels of p-STAT, STAT, and p53 (a), p21, CDK1, and cyclin B1 (b) as well as CDK1 activity (c) as described in Materials and Methods. Each value is mean ± S.D. of three experiments; a, *P* ≤ 0.05, is significantly different compared with control (0 nM); b and c, *P* ≤ 0.05, reveal significantly difference compared with 250 and 500 nM of cucurbitacin E, respectively (one-way ANOVA followed by Bonferroni's test for multiple comparisons).

To investigate the molecular mechanisms of cucurbitacin E-induced G_2_/M phase arrest in T24 cells, cucurbitacin E-treated cells determined the G_2_/M phase-modulated relative protein levels. Results from western blotting and CDK1 activity were shown in Figure 3. Our data indicated that the level of p53 was increased, and the level of p-STAT3 was decreased, but the level of STAT3 was not altered (Figure 3(a)) in cucurbitacin E-treated T24 cells. The level of p21 was upregulated, but the levels of CDK1 and cyclin B were downregulated, leading to induce G_2_/M phase arrest. The CDK1 activity also was inhibited by cucurbitacin E and this effect is a dose-dependent manner (Figure 3(c)). Based on these results, we suggest that cucurbitacin E-increased G_2_/M phase arrest in T24 cells might be involved in STAT3/p53/p21 signaling.

### 3.4. The Effects of Cucurbitacin E on Morphological Observation and Apoptosis in T24 Cells

**Figure 4:**
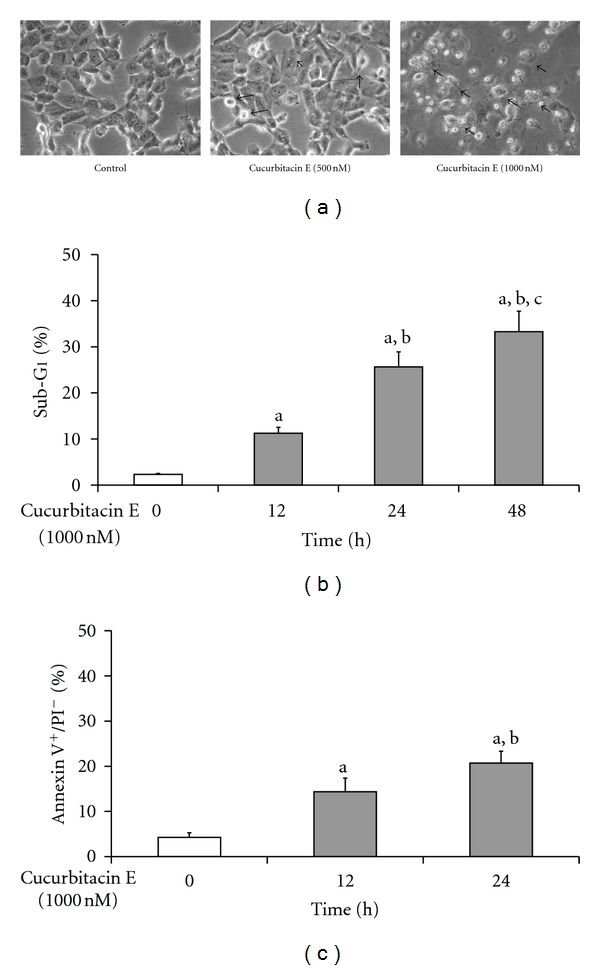
The effects of cucurbitacin E on the apoptotic death of T24 cells. Cells at a density of 2 × 10^5^ cells/well were placed in 12-well plates then were treated with 500 and 1000 nM of cucurbitacin E for examining the morphological changes (a). Cells were incubated with 1000 nM cucurbitacin E for different time periods (12, 24, and 48 h), and then cells were harvested for evaluating the sub-G1 phase (apoptosis) by flow cytometry (b). Cells were exposed to 1000 nM cucurbitacin E for 12 and 24 h, and then cells were harvested for determining the annexin V positive/PI negative (apoptotic cells) as described in Materials and Methods. The results are shown as a mean ± SD (*n* = 3); a is significantly different (*P* ≤ 0.05) compared to control; b and c represent significant difference (*P* ≤ 0.05) compared with 1000 nM cucurbitacin E for 12 and 24 h, respectively, by one-way ANOVA followed by Bonferroni's test for multiple comparisons.

Cells were exposed to 0 (control), 500, and 1000 nM of cucurbitacin E for 48 h, and then examined and photographed by a phase-contrast microscope. Moreover, cells were treated with 1000 nM cucurbitacin E for 0, 12, 24, or 48 h and then assayed the percentage of sub-G1 phase by flow cytometry. The results shown in Figure 4(a) indicated that cucurbitacin E induced morphological changes such as membrane blebbing, reduction in cell volume, and these effects are dose-dependent manners (Figure 4(a)).  Figure 4(b) also shows that cucurbitacin E induced sub-G1 phase (apoptosis) in cell cycle distribution that indicated that cucurbitacin E induced apoptosis, and this effect is a time-dependent manner. Apoptotic cells were also confirmed by Annexin V/PI staining assay in cucurbitacin E-treated T24 cells as seen in Figure 4(c).

### 3.5. The Effects of Cucurbitacin E on Protein Levels of Caspase-3, -8, and -9 in T24 Cells

**Figure 5:**
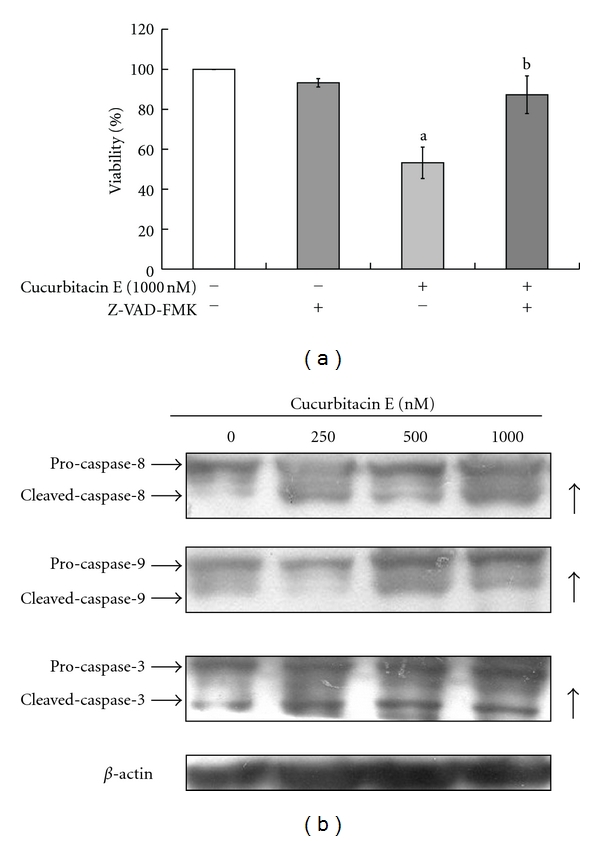
The effects of a general caspase inhibitor (Z-VAD-FMK) on cucurbitacin E-induced cell viability and caspase cascades protein levels on T24 cells. Cells were pretreated with 10 *μ*M Z-VAD-FMK and then exposed to 1000 nM cucurbitacin E for 48 h. Cells were harvested and determined for percentage of viable cells (a) and for evaluating the protein levels of caspase-8, -9, and -3 (b) as described in Materials and Methods. Columns: mean (*n* = 3); bars: SD. a, *P* ≤ 0.05, is significantly different compared with untreated control; b, *P* ≤ 0.05, shows significant difference compared with 1000 nM cucurbitacin E treatment group by one-way ANOVA followed by Bonferroni's multiple comparison test.

To investigate if cucurbitacin E induces apoptosis in T24 cells through caspases-dependent pathway, cells were pretreated with a general caspase inhibitor (Z-VAD-FMK) and then exposed to cucurbitacin E for 48 h treatment. Results indicate that Z-VAD-FMK is able to protect against cucurbitacin E-reduced cell viability in T24 cells. Also, our results from western blotting indicated that the levels of active form of caspase-3, -8, and -9 were stimulated in cucurbitacin E-treated T24 cells (Figure 5(b)). This study suggests that cucurbitacin E-induced apoptosis of T24 cells is mediated *via* caspase cascade-dependent pathway.

### 3.6. The Effects of Cucurbitacin E on Mitochondrial Membrane Potential (ΔΨ_*m*_) of T24 Cells

**Figure 6:**
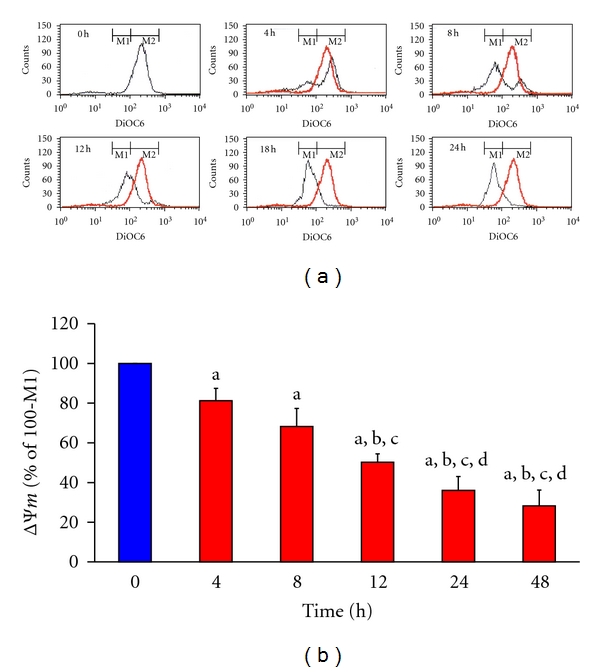
The effects of cucurbitacin E on mitochondrial membrane potential (ΔΨ_*m*_) in T24 cells. Cells at a density of 2 × 10^5^ cells/well placed in 12-well plates were treated with 1000 nM cucurbitacin E for 0, 4, 8, 12, 24, and 48 h and then harvested for measuring the level of ΔΨ_*m*_ as described in Materials and Methods. (a) The representative of profile of ΔΨ_*m*_, and (b) the levels of ΔΨ_*m*_ quantified form BD CellQuest Pro software. Each experiment was done with triple sets: a, *P* ≤ 0.05, shows significant difference compared with 0 h treatment; b, c, and d, *P* ≤ 0.05, are significantly different compared to cucurbitacin E treatment for 4, 8, and 12 h, respectively, by one-way ANOVA followed by Bonferroni's multiple comparison test.

To investigate whether mitochondrial dysfunction is involved in cell apoptosis induced by cucurbitacin E in T24 cells, cells were exposed to cucurbitacin E for 0, 4, 8, 12, 24, and 48 h and subsequently harvested for determination of the level of ΔΨ_*m*_ by flow cytometry. Results shown in Figures 6(a) and 6(b) indicated that cucurbitacin E decreased the level of ΔΨ_*m*_ and this effect is a time-dependent manner. 

### 3.7. The Effects of Cucurbitacin E on Apoptosis-Associated Protein Levels in T24 Cells

**Figure 7:**
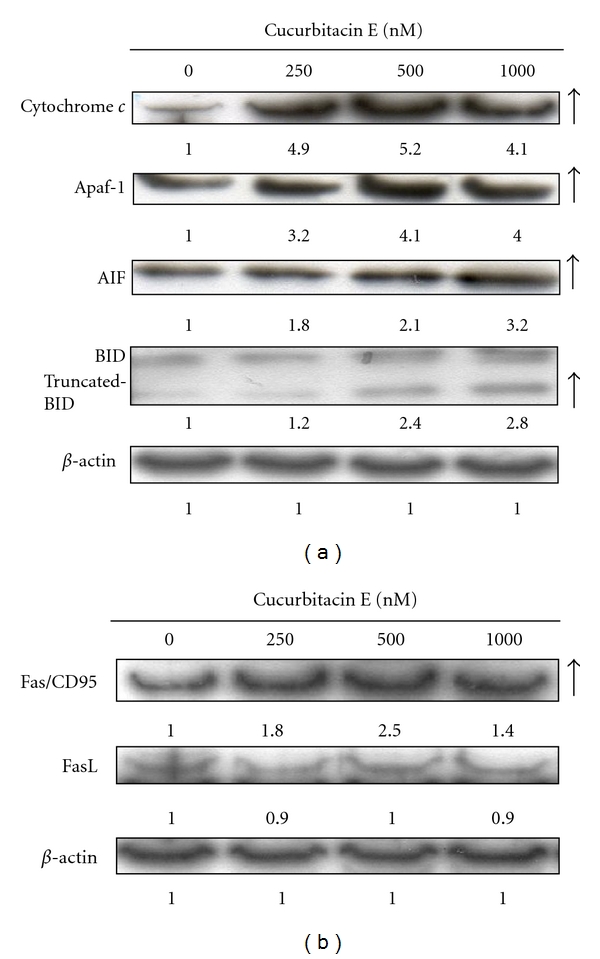
The effects of cucurbitacin E on the apoptosis-associated proteins in T24 cells. Cells (1 × 10^6^/well) seeded into 6-well plates were treated with 1000 nM cucurbitacin and were treated with different concentrations of cucurbitacin E for 48 h and then harvested for western blotting to examine the protein levels of cytochrome *c*, Apaf-1, BID, and truncated BID (a), Fas and FasL (b) as described in Materials and Methods.

It is well known that mitochondria-mediated activation of caspase-9 and caspase-3 involved the releases of cytochrome *c *and AIF [37, 38]. The treatment with cucurbitacin E increased the levels of cytochrome *c*, Apaf-1, AIF, truncated BID (Figure 7(a)), and Fas (Figure 7(b)) in cucurbitacin E-treated T24 cells. These data suggest that the increased levels of Fas/CD95, cytochrome *c*, Apaf-1, and truncated BID led to the activation of caspase-8, caspase-9, and caspase-3 (Figure 5(b)). Hence, cucurbitacin E-provoked apoptosis in T24 cells is mediated through the Fas/CD95 and mitochondria-dependent pathways.

## 4. Discussion

It is well known that the resistant to current therapies is one of the poor prognostic outcomes of human bladder cancer and it is the leading cause of cancer-related death [39, 40]. Currently, the successful treatment with chemotherapeutic agents is based on these agents to trigger cell death in tumor cells [41–43]. Until now, investigators have been focusing on the novel inducers of apoptosis to provide a new therapeutic approach for cancer patients and some of the compounds for treatment of cancer are from natural products [44–46].

Results showed that cucurbitacin E induced G_2_/M phase arrest (Figure 2) in T24 cells. The protein levels of phospho-STAT3, CDK1, and cyclin B were decreased, and the levels of p53 and p21 were increased in cucurbitacin E-treated T24 cells in a time-dependent manner (Figures 3(a) and 3(b)). Furthermore, curcumin E also inhibited the CDK1 activity in T24 cells (Figure 3(c)). It is reported that activated STAT3 is formed through downregulating p53 level and affects the p53 promoter in *in vitro* and *in vivo* studies [47]. Moreover, CDKs regulate the cell-cycle progression in mammalian cells [48, 49]. In the G_2_/M phase progression which is regulated with CDK1 and CDK2 kinases that are activated primarily in association with cyclins A and B [50]; it is reported that distributing cell-cycle progression by alterations in cell cycle-related protein expression plays important roles in the proliferation of cancer cells [50, 51]. Therefore, we suggest that alterations in STAT3/p21/p53 signaling and G_2_/M phase-associated protein levels occurred in cucurbitacin E-treated T24 cells. This is also in agreement with other reports addressing that inhibitory effect of STAT3 level on cucurbitacin E and triterpene-derived natural products treated human tumor cells and umbilical vascular endothelial cells *in vitro* [9, 11, 51]. 

Our results also showed that cucurbitacin E induced apoptotic death of T24 cells and this effect is a dose- and time-dependent response (Figure 4). Apoptosis is a programmed cell death which is a multiple regulated process which can be divided into the caspases and mitochondrial signaling pathways [52, 53]. Cucurbitacin E treatment in T24 cells promoted the activations of caspase-8, -9, and -3 in a dose-dependent manner (Figure 5(b)). Otherwise, cells were pretreated with a general caspase inhibitor (Z-VAD-FMK) and exposed to cucurbitacin E, leading to increase the percentage of viable cells when compared to the cucurbitacin E-treated only cells (Figure 5(a)). The data indicated that the activation of caspase cascade is involved in cucurbitacin E-induced cell death

 Result from Figures 6(a) and 6(b) indicated that cucurbitacin E decreased the level of ΔΨ_*m*_ in a time-dependent response. Bcl-2 family proteins (antiapoptotic and proapoptotic proteins) have been reported to regulate cytochrome *c *release from mitochondria [53, 54]. Our results also showed that cucurbitacin E promoted the level of cytochrome *c* (released from mitochondria) (Figure 7(a)). Cucurbitacin increased the levels of truncated BID which is related to the dysfunction of mitochondria (Figure 7(a)). Figure 7(a) also shows an increase in the level of AIF which is released from mitochondria. Based on these observations, we may suggest that cucurbitacin-induced apoptosis of T24 cell is carried out through the Fas/CD95 and mitochondrial signaling pathways.

**Figure 8:**
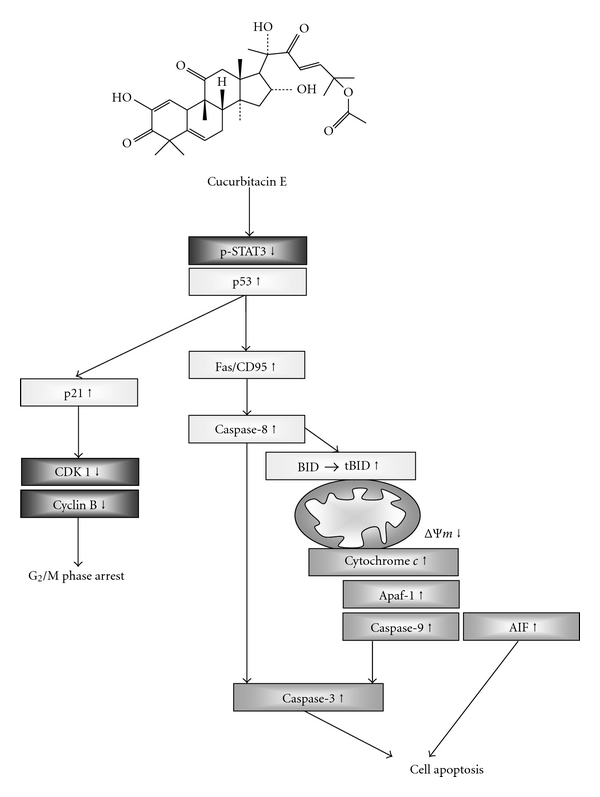
The possible signaling pathways for cucurbitacin E-induced G_2_/M phase arrest through STAT3/p53/p21 signaling and apoptosis *via* Fas/CD95 and mitochondria-dependent pathways in human bladder cancer T24 cells.

In conclusion, cucurbitacin E decreased the percentage of viable T24 cells through the cell cycle arrest and induction of apoptosis in human bladder T24 cancer cells* in vitro*. Cucurbitacin E-induced G_2_/M phase arrest was associated with the inhibitions of phosphorylation STAT3, promotion of p53 and p21, and reduction of CDK1 and cyclin B. Cucurbitacin E-induced apoptosis was accompanied with upregulation of Fas/CD95, decrease the level of ΔΨ_*m*_ and then led to cytochrome *c* release from mitochondria and promoted the activations caspase-8, caspase-9, and caspase-3, leading to apoptosis of T24 cells. The proposed signaling pathways can be seen in Figure 8.
